# Surgical management of ancient retroperitoneal schwannoma

**DOI:** 10.1016/j.amsu.2022.103934

**Published:** 2022-06-06

**Authors:** Abdelhakim Harouachi, Nabil Khomsi, Houssam Aabdi, Nada Akouh, Tariq Bouhout, Amal Bennani, Tijani EL. Harroudi, Badr Serji

**Affiliations:** a Surgical Oncology Department, Regional Oncology Center, Mohammed VI University Hospital, Oujda, Morocco; b Mohammed First University Oujda, Faculty of Medicine and Pharmacy Oujda, Oujda, Morocco; c Department of Pathology, Mohammed VI University Hospital, Oujda, Morocco

**Keywords:** Retroperitoneal Schwannoma, Complete resection, Retroperitoneal mass

## Abstract

Schwannomas are benign and rare entities of peripheral nerve sheath origin. The clinical presentation depends the size of tumor, and they may cause symptoms of abdominal pain, urinary difficulties, anemia, hematuria, and abdominal mass as a result of the pressure of the tumor to the adjacent structures. The diagnosis can only be established by immunohistochemical study. Complete surgical clearance remains the mainstay of treatment.

We report the case of a 35-year-old female patient consulted for chronic abdominal pain. Abdominal ultrasound and abdomino-pelvic CT scan identified a well-delineated, heterogenous retroperitoneal mass developed in anteraortocaval region measuring 55 × 65 × 88 mm. The lesion seemed to repress the inferior vena cava, and enhanced with contrast administration. The histopathological panel confirmed the diagnosis of retroperitoneal schwannoma.

## Highlights

• Schwannomas are benign and rare entities of peripheral nerve sheath origin.
• They do not lead to an invasion of the adjacent structures; however the compression and displacement of the retroperitoneal organs are marked as complications of increasing in size of these entities.
• The total excision is the sole treatment for retroperitoneal schwannoma.

## 1 Introduction

Schwannomas, also called Neurilemmomas, are types of peripheral nerve tumors, benign disease, mostly located in the head and the neck, which are arising from Schwann cells of the nerve sheath. These tumors contribute to 0.75–2.6% of all retroperitoneal neoplasms, and the majority of patients are between 20 and 50 years old with a female predominance [1,2,6]. These tumors appear to arise in the retroperitoneum involving usually the paravertebral space or presacral region [3]. Retroperitoneal schwannomas (RSs) generally have no infiltrating or metastatic potential. They do not lead to an invasion of the adjacent structures; however the compression and displacement of the retroperitoneal organs are marked as complications of increasing in size of these entities [4].

The diagnosis can only be established by histological and immunohistochemical study. The total excision is the sole treatment [1,2]. We report a case of an ancient retroperitoneal schwannoma (RS) in a 39-year-old female patient. This work has been reported in line with the SCARE 2020 Guideline [5].

## 2 Case presentation

We present a 39-year-old female (gravida 2, parity 2) with no significant past medical or surgical history had presented in 2019 to our department chiefly complaining of chronic abdominal pain of five months duration with a severity of 5 in 10. The pain has been mainly located in the epigastrium and umbilical region. It has been radiating to the back and has been not associated with any aggravating or mitigating factors. Notably absent were symptoms of nausea, vomiting, fever, bloating, and change in bowel habit or weight loss. There has been no history of cancer in her family, without reference to using drugs, alcohol or tobacco. She had no psychological history.

Computed tomography (CT) of the abdomen and pelvis has revealed a well-delineated, heterogenous retroperitoneal mass developed in anteraortocaval region measuring 55 × 65 × 88 mm. The lesion seemed to repress the inferior vena cava, and enhanced with contrast administration. No metastases were found. The level of serum tumor marker, were within the normal range.

Based on the clinical and radiological findings, the patient underwent a midline laparotomy; intra-operative exploration has revealed a large retroperitoneal mass, difficult to access to the presence of several mesenteric lymphadenopathies. A lymph node dissection was performed.

The Pathological examination has revealed that the lymph nodes were reactive without a specific element of malignancy. The patient was lost to follow-up.

**Fig. 1:**
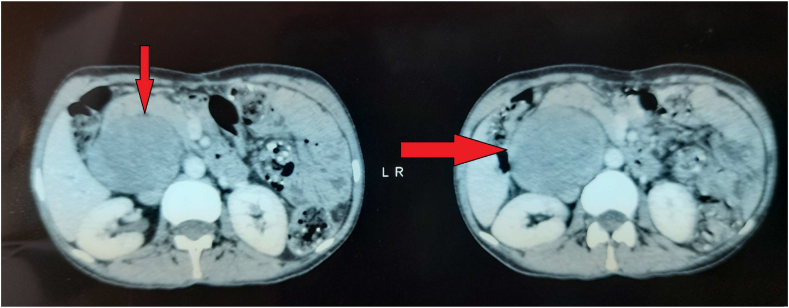
Abdomino-pelvic computed tomography demonstrates a retroperitoneal mass developed in anteraortocaval region measuring 55 × 65 x 88 (arrow).

In September 2021, the patient presented herself in our department with complaints of distention and abdominal pain. Vital parameters and all laboratory investigations, including blood exam, kidney function and urinalysis, were within normal limits. Abdominal computed tomography scanning was performed, revealed an oval mass increased in size (68 × 74 x 98 compared to 55 × 65 x 88) (Fig. 1). The case was analyzed by a multidisciplinary committee and it was decided that second look laparotomy was considered to be the best option.

Due to the risk of bleeding and the unavailability of blood, a surgical biopsy of the mass with Tru-cut® was performed, and pathology report confirmed the diagnosis of a retroperitoneal Schwannoma.

The surgery for the mass removal after obtaining the patient's consent was performed. The procedure was performed by a professor of surgery with over 15 years of surgical experience in an academic hospital. Under general anesthesia, the patient was placed in the supine position. The patient underwent laparotomy with midline abdominal incision where multiple adhesions were seen. After releasing the adhesions, the lesion was seen on anteraortocaval region (Fig. 2a and Fig. 2b). We carefully were dissected and completely excised the mass from the edges of the aorta and vena cava, so as not to damage the adjacent tissues. A suction drainage tube was placed in the recto vesical pouch. Total operative time was 140 min, with an estimated blood loss of 50 ml. Macroscopically, the mass was poorly circumscribed and haemorrhagic, with a diameter of 10 × 8 × 5.5cm and weight of 260g.

**Fig. 2a:**
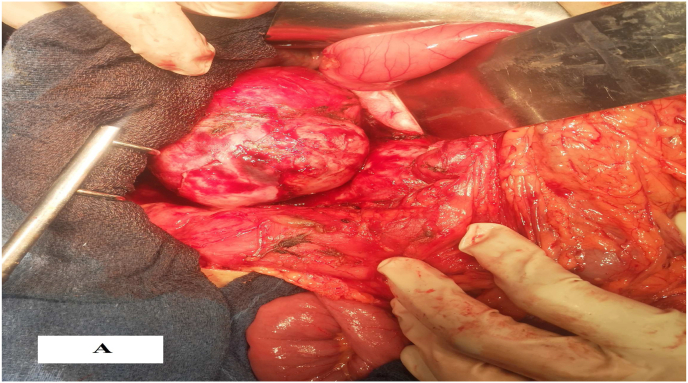
Intra-operative view of a tumor.

**Fig. 2b:**
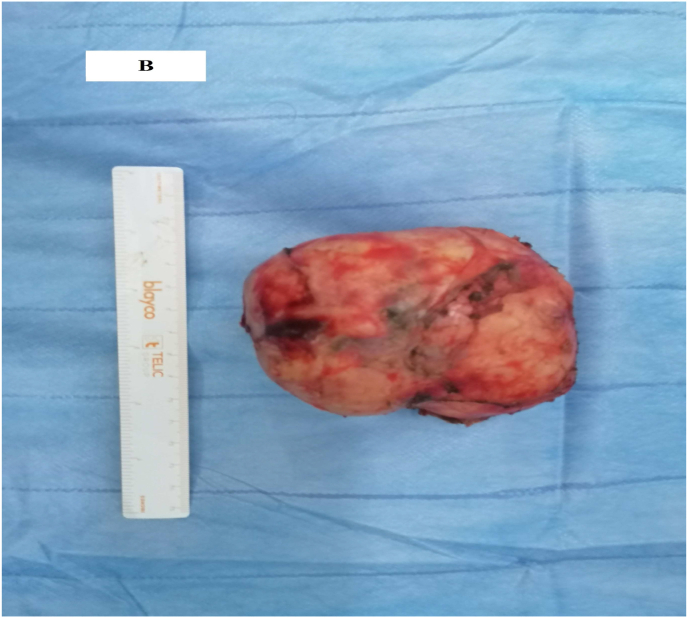
Image of the mass after its removal.

Pathological examination of the specimen has a benign tumor proliferation arranged essentially in a single area, ANTONI B where the cellularity is loose and made up of regular fusiform cells without atypia or mitosis. This proliferation arranged in a wavy fashion. Tumor cells express PS100 strongly, suggesting that the tumor was a schwannoma (Fig. 3a and Fig. 3b).

**Fig. 3a:**
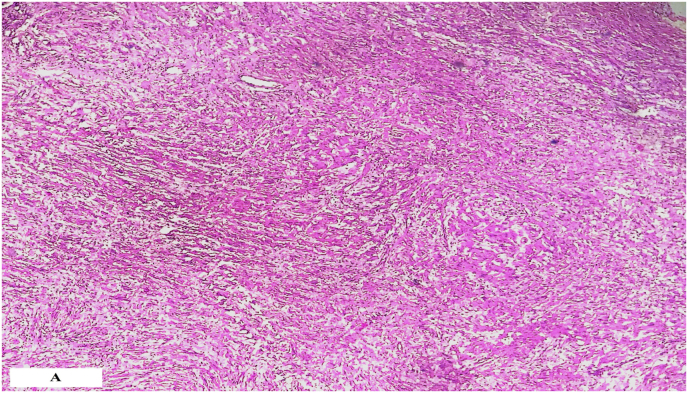
Photomicrograph showing a benign tumor proliferation arranged in a wavy fashion.

**Fig. 3b:**
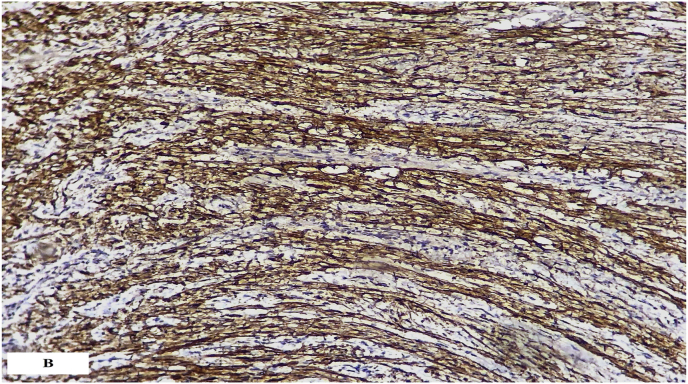
Immunohistochemical analysis showed diffuse strong PS100 positivity.

After an uneventful postoperative course, the patient was discharged 4 days after surgery. Presently, the patient is doing well, without recurrence of the tumor during the five months follow-up examination.

## 3 Discussion

Schwannomas are benign and rare entities of peripheral nerve sheath origin with predominance usually in young to middle-aged adults, rarely degenerate into a malignant form [1,4]. These neurogenic tumors can be found anywhere but most commonly found in the upper extremities or in cranial and peripheral nerves in the head and neck [6]. They usually affect two women as often as men [8]. The etiopathogenesis of RSs is not completely understood, however 5%–18% cases have been related to von Recklinghausen's disease [7].

The most common symptoms associated with retroperitoneal schwannoma are vague because of the flexible feature of the retroperitoneum. Patients with RSs can present asymptomatically in the early stages of its development. The clinical presentation of these entities depends the size of tumor, and they may cause symptoms of abdominal pain, urinary difficulties, anemia, hematuria, and abdominal mass as a result of the pressure of the tumor to the adjacent structures [1,4,8]. Radiological exploration relies mainly on computed tomography scan and Magnetic Resonance Imaging MRI. They are the reference modalities for evaluating the size and the origin of the mass, and the involvement of the surrounding organs. Benign RSs are manifested on CT scan by a well-defined and homogeneous mass with minimal enhancement [2,9]. The imaging tool MRI suggests the diagnosis of neurogenic tumors by the presence of a hypointense center with a hyperintense periphery (the target sign), however there is no specific radiological features of RSs [10,11].

The immunohistochemical study is a valuable tool for validating the diagnosis of RSs based on positive immunostaining for S-100, vimentin and NSE, and negative labelling for desmin, SMA, HHF35, CD34 and CD117 [1,2].

The best and optimal treatment for retroperitoneal schwannoma is a complete resection. This option is not always possible due to extend to neighboring anatomical vital structures [1,2]. In our case, the surgery was a challenge, but the complete excision was performed. The laparoscopic approach is inappropriate due to the lack of courageous results [12,13].

In summary, the surgical resection is an effective treatment modality for retroperitoneal schwannomas, and should be performed by and an experienced surgeon to avoid intra-operative vascular lesions. The preoperative diagnosis remains a challenge for the clinician.

## Ethical approval

No ethical approval necessary.

## Sources of funding

The author(s) received no financial support for the research, authorship and/or publication of this article.

## Registration of research studies

Our paper is a case report; no registration was done for it.

## Patient consent

Written informed consent was obtained from the patient for publication of this case report and accompanying images. A copy of the written consent is available for review by the Editor-in-Chief of this journal on request.

## Provenance and peer-review

Not commissioned, externally peer reviewed.

## Declaration of competing interest

The authors declare that they have no conflict of interest.

## Funding information

This research has not been supported by any private or corporate financial institutions nor has any grant been received for this study.

## Guarantor

Harouachi Abdelhakim.

## CRediT authorship contribution statement

**Abdelhakim Harouachi:** Have written the article, have consulted the patient, prescribed all of the tests and prepared the patient for surgery and participated in the surgery. Dr. **Nabil Khomsi:** have helped writing the article, data collection. Dr. **Houssam Aabdi:** have helped writing the article, data collection. Dr. **Tariq Bouhout:** (oncology surgery professor): supervised the writing of manuscript. Pr. **Amal Bennani:** (Pathology professor): confirm the histological diagnosis. Pr. **Tijani EL. Harroudi:** (oncology surgery professor): supervised the writing of manuscript. Pr. **Badr Serji:** (oncology surgery professor): have supervised the writing of the paper, and had been the leader surgeon of the case.

## Declaration of competing interest

The authors declared no potential conflicts of interests with respect to research, authorship and/or publication of the article.
